# Individual Consistency and Phenotypic Plasticity in Rockhopper Penguins: Female but Not Male Body Mass Links Environmental Conditions to Reproductive Investment

**DOI:** 10.1371/journal.pone.0128776

**Published:** 2015-06-01

**Authors:** Nina Dehnhard, Marcel Eens, Laurent Demongin, Petra Quillfeldt, Maud Poisbleau

**Affiliations:** 1 University of Antwerp, Department Biology—Ethology, Campus Drie Eiken, Universiteitsplein 1, Antwerp (Wilrijk), Belgium; 2 Max Planck Institute for Ornithology, Department of Migration and Immuno-Ecology, Am Obstberg 1, Radolfzell, Germany; 3 University of Konstanz, Department of Biology, Konstanz, Germany; 4 Justus-Liebig University Gießen, Department of Animal Ecology & Systematics, Heinrich-Buff-Ring 38, Gießen, Germany; Phillip Island Nature Parks, AUSTRALIA

## Abstract

In marine habitats, increasing ocean temperatures due to global climate change may distinctly reduce nutrient and consequently food availability for seabirds. Food availability is a known driver of body mass and reproductive investment in birds, but these traits may also depend on individual effects. Penguins show extreme intra-annual body mass variation and rely on accumulated body reserves for successful breeding. However, no study so far has tested individual consistency and phenotypic responses in body mass and reproductive investment in this taxon. Using a unique dataset on individually marked female and male southern rockhopper penguins (*Eudyptes chrysocome chrysocome*) across six years, we investigated 1) the individual consistency in body mass (measured at egg laying), body condition and reproductive investment across years, subsequently 2) identified the best-explanatory temperature-related environmental variables for female and male body mass, and 3) tested the effect of female and male body mass on reproductive investment. Body mass, body condition and reproductive investment were all highly repeatable. As body condition should control for the structural size of the birds, the similarly high repeatability estimates for body mass and body condition suggested that the consistent between-individual body mass differences were independent of structural size. This supported the use of body mass for the subsequent analyses. Body mass was higher under colder environmental conditions (positive Southern Annular Mode), but the overall phenotypic response appeared limited. Reproductive investment increased with female but not male body mass. While environmental effects on body mass in our study period were rather small, one can expect that ongoing global climate change will lead to a deterioration of food availability and we might therefore in the long-term expect a phenotypical decline in body mass and reproductive investment.

## Introduction

Currently, climate change is seen as the most wide-ranging and dangerous threat for plants and animals in the 21st century and beyond [1]. Climate change scenarios predict that increasing CO_2_ concentrations lead to globally rising air and ocean temperatures, acidification of the marine environment and an increase in climate variability ([2] and references therein). In the marine environment, the water column undergoes a shallower and more stable stratification under higher temperatures, resulting in a reduced availability of macronutrients for primary producers in the light-exposed upper zone of the ocean [3]. As a consequence, ocean productivity decreases [3] and changes in the composition of the food web occur [4]. Temperature changes can therefore affect ocean productivity and consequently availability of food in space and time [5, 6]. Such changes in food availability may affect body mass of animals [7–9] and/or parental investment into reproduction [10, 11]. Both responses reflect a form of phenotypic plasticity (i.e. the ability of a single genotype to modify its phenotype in response to short-term environmental conditions; [12]), a trait that appears particularly important for long-lived animals to adapt to the consequences of global climate change [13].

Especially in income breeders, i.e. animals that acquire the necessary energy for breeding concurrently [14], environmentally driven food availability may directly affect reproductive investment of parents. On the other hand, in capital breeders, i.e. animals that accumulate energy reserves for breeding before the actual reproductive period [14, 15], food availability should affect reproductive investment indirectly, through the link of body mass. In fact, a positive effect of female body mass or body condition (i.e. body mass corrected for body size, thus reflecting energy reserves) on egg and/or offspring mass has been described in various taxonomic groups [16–18]. The same relationship, albeit less consistently, has been shown for males [17, 19]. Mechanistically, male body mass (as well as other sexual signals such as male colouration or song) should indicate “mate quality”, with the consequence of females investing more energy into eggs when they can expect a higher contribution to parental care by their male partner [20–22]. Likewise, individual differences in body mass (or body condition) over time may result in differential reproductive investment among these individuals [23, 24].

Considering that body mass may thus affect reproductive investment (linked to life-time reproductive success; [25]), the effects of global climate change may go beyond a reduction in body mass and reproductive investment as a phenotypically plastic response. In the long term, the effect of decreased food availability on body mass due to increasing temperatures may also have consequences for population dynamics and the conservation status of species.

Penguins show extreme intra-annual variation in their body mass due to extended fasting periods during incubation and moult, when they may lose more than 40% of their body mass [26]. Both partners invest heavily into parental care and therefore need to acquire the necessary energy reserves prior to breeding, as they will abandon reproductive duties when reaching a critical minimum threshold body mass [27, 28]. Inter-annual as well as between-individual differences in body mass at arrival in colonies may therefore be directly linked to breeding success [29–31]. Consequently, individual body mass in both sexes may be a visual indicator of the future ability to attend the nest and thus the birds’ investment into reproduction. Poisbleau et al. [32] indeed showed that females adapted resource-allocation according to their male partners’ mass. That said, and considering the extreme intra-annual variation, there is still a complete lack of information on whether body mass and reproductive investment are consistent in individual penguins across time. However, such an individual-based approach with data collected across several years is critically important to differentiate between environmental and individual effects and assessing the level of phenotypic plasticity when analysing variation in body mass and reproductive investment.

Southern rockhopper penguins (*Eudyptes chrysocome chrysocome*) are one of the smallest species of penguins [33] and ideal to study the phenotypic plasticity in body mass and reproductive investment as a response to their environment. They mainly feed on low trophic level prey (e.g. krill, small fish and squid; [34]). This makes them sensitive to changes in environmental conditions that affect local primary productivity. As other crested penguins (genus *Eudyptes*), they are long-lived, monogamous and exhibit a high fidelity to both nest-site and partner, usually attempting to breed every year [35, 36], which enabled us to study the same individuals across several years. Furthermore, they are typical capital breeders [15] and therefore acquire the necessary energy reserves for reproduction before and during their migration to breeding sites. Thus, if environmental conditions affect food availability and body mass, this should also be visible in the reproductive investment.

Rockhopper penguins lay a two-egg clutch, with chicks from first-laid (A-)eggs hatching about one day after the chicks from second-laid (B-)eggs [37]. Maternal investment for B-eggs is higher, as these are on average 28% larger and heavier than A-eggs [37, 38]. A-chicks often die in the first days after hatching, but occasionally parents fledge both chicks [37, 39].

The marine foraging habitat of the southern rockhopper penguin is influenced by the cold, nutrient-rich Falkland Current that originates north of the Antarctic Peninsula [40, 41]. This area has undergone one of the strongest warming trends worldwide [42, 43], which is reflected in the advancement of breeding, demographic responses and distribution shifts in local Antarctic penguin species (e.g. [44, 45]). Effects of this warming trend on southern rockhopper penguins therefore appear very likely in the long-term. Considering that the species is currently listed as vulnerable [46], predictions on how environmental changes affect adult body mass and consequently reproductive investment will be important for conservation actions.

Using a dataset of individually marked females and males, their body mass at clutch initiation, and egg mass across six years, we 1) tested for the repeatability of body mass and egg mass within individuals to understand whether individuals may be consistent in these traits across several years. We also tested for the repeatability of body condition, in order to assess the potential effect of structural body size on repeatability of body mass. Subsequently, 2) we tested for the expected effect of temperature-related environmental conditions on body mass and consequently 3) of body mass on egg mass. We therefore identified which one of several à-priori chosen temperature-related environmental variables explained variation in body mass across years best, and then tested the following predictions: A) As lower temperatures generally imply higher primary productivity and food availability in the marine ecosystem, we expected body mass of both sexes to be higher under such colder conditions; B) Heavier birds can afford to invest more energy into reproduction (besides egg laying also subsequent feeding of the offspring; e.g. [47]). We therefore expected egg mass to increase with parental (both female and male) body mass. Due to the pronounced egg-mass dimorphism, we considered A- and B-egg mass separately.

## Methods

### Ethics Statement

The study was performed according to Belgian and Flemish law and was approved by the ethical committee on animal experimentation (ECD, ID number: 2011/44). All work was conducted under research licenses granted by the Environmental Planning Department of the Falkland Islands Government. The field site is owned and protected by the New Island Conservation Trust. Initial marking of birds with passive integrative transponders did not lead to any adverse effects [48]. Nest checks and weighing of adults and eggs did not cause any desertion from breeding activity or mortality.

### Field Methods

Fieldwork was done in the “Settlement Colony” on New Island, Falkland Islands / Islas Malvinas (51°43’S, 61°17’W) between 2006 and 2014. The colony held about 5,700 breeding pairs during the first breeding season in 2006/07, and 8,200 breeding pairs during the last breeding season in 2013/14. More specifically, we worked in one part of the Settlement Colony that includes almost one quarter of the nests and is representative in vegetation and topography of the entire colony. In this part, starting in 2006/07, we gradually marked more than 800 adult birds (i.e. three years of age or older) subcutaneously with passive integrated transponders (PITs; 23 mm long, glass-encapsuled, TIRIS, Texas Instruments, USA; [48]). The sex of the birds was determined from a combination of morphological and behavioural observations; males are larger than females and both have a fixed pattern of nest attendance and incubation shifts [49]. The breeding cycle of southern rockhopper penguins has been described previously [37]. Briefly, males arrive in breeding colonies in the first week of October, followed by the females a few days later. Both males and females stay ashore and fast during the entire courtship and egg laying period and the first incubation shift. Males leave colonies for a ca. 10-day foraging trip in the middle of November, while females incubate eggs until males return and then leave the colony themselves for foraging.

In the framework of an on-going project on maternal investment [32, 37, 50], we collected data on egg mass and female and male body mass across multiple breeding seasons (2006/07–2007/08, 2009/10–2010/11 and 2012/13–2013/14). We visited the colony daily from at least mid-October onwards to follow the egg laying of focal females equipped with a transponder. Transponders were read with a hand-held antenna (Allflex RS320). Clutches were initiated between 25 October and 9 November in all years. We weighed both A- and B-eggs to the closest 0.1 g using a digital balance (Kern CM 320-1N; Kern & Sohn, Germany) on the day when they were first observed. As incubation in rockhopper penguins typically does not start before clutch completion [26], the A-eggs were not incubated at all and the B-eggs were not incubated for longer than 24 h at weighing. We therefore assumed that embryo development and (potential) change in egg mass (see [50]) had not yet begun.

We captured males and females on their nests, covered their head to minimize stress and weighed them to the nearest 20 g with an electronic balance following Poisbleau et al. [49]. We further measured flipper length (using a ruler to the nearest mm), bill depth and bill length (to the nearest 0.1 mm, using callipers). Birds were released after approximately 10 minutes and returned to their partner on the nest. The general procedure was to capture females on their clutch initiation date (= A-egg laying date) and males 5–8 days later. We chose this standardized procedure instead of capturing both partners on the same day in order to minimize the immediate disturbance. However, because of logistic reasons, some females were captured 22–12 days before (N = 36 events; 10%) or 1–23 days after (N = 110 events; 30%) clutch initiation. In order to enable comparisons, we therefore estimated their body mass at clutch initiation by applying a correction by the effect of fasting on body mass. We used 25 females repeatedly (2 or 3 times) weighed in 2010 between their arrival at the colony and the end of their first fasting shift, i.e. the one including egg laying. Female body mass was corrected by removing A-egg mass for captures before clutch initiation and by adding B-egg mass for captures after B-egg laying. We performed a linear mixed effect model with bird identity as random factor and capture date as only explanatory variable. We extracted the estimates (f(x) = 4401.2–33.3*x; marginal R^2^ = 0.917) and consequently assumed a linear body mass decrease by 33.3 g per fasting day. We followed the same procedure to estimate male body mass at clutch initiation (N = 48 males repeatedly weighed in 2010; f(x) = 4453.8–38.6*x; marginal R^2^ = 0.793 and body mass decrease of 38.6 g per fasting day). We therefore used these values to back-calculate body mass at clutch initiation for 146 females and all males.

In the present study, we included only the nests for which we obtained A-egg mass, B-egg mass, female and male body mass. This resulted in a database of 366 records (between 42 and 86 nests per breeding season) from 202 different females and 222 different males (numbers differed between sexes due to few cases of divorces) represented on average 1.81 ± 1.23 S.D. (min. 1, max. 5) and 1.65 ± 1.07 S.D (min. 1, max. 5) times, respectively across six breeding seasons.

### Environmental Variables

We evaluated the effect of three different environmental variables on adult female and male body mass: the two broad-scale climatic indices Southern Annular Mode (SAM) and Southern Oscillation Index (SOI) as well as local sea surface temperature anomaly (SSTA). All three variables are temperature-related and we here also consider them as potential proxies for food availability, as a direct quantification of food availability in the ocean is problematic. SAM is the dominant mode of atmospheric variability in the Southern hemisphere, with distinct effects on wind patterns and sea surface temperatures [51]. SOI (also referred to as El Niño Southern Oscillation or ENSO) is defined as the air-pressure difference between the mid-Pacific (Tahiti) and west-Pacific (Darwin). Both of these broad-scale climatic indices have effects on sea surface temperatures in the South Atlantic Ocean, with positive SAM and SOI indices coupled to lower surface temperatures [52, 53]. Local SSTA represent a different spatial scale and thus reflect environmental conditions close to the colony. All three variables have previously shown to affect either breeding biology or population dynamics of other seabird species, including (rockhopper) penguins [54–60].

Similarly to Lynch et al. [45], we averaged SAM, SOI and SSTA for the three-month period from August through to October. Monthly SAM data were downloaded from the British Antarctic Survey (http://www.nerc-bas.ac.uk/icd/gjma/sam.html), and monthly SOI data were obtained from the University Center for Atmospheric Research Climate Analysis Section Data Catalogue (http://www.cgd.ucar.edu/cas/catalog/climind/SOI.signal.ascii). For local SSTA (in°C), we selected a 2° grid in the west of New Island (50–52°S, 61–63°W). This area is known to be the major foraging location of this population of southern rockhopper penguins during the breeding season [61, 62]. Due to their migratory behaviour in winter, it is, however, not proven yet whether the penguins utilize this area shortly before arrival to the breeding sites in spring. Monthly SSTA data were based on the difference between monthly sea surface temperature and the long-term monthly average (data from 1971 to 2000). These data were obtained from the National Oceanic and Atmospheric Administration (NOAA: http://iridl.ldeo.columbia.edu/SOURCES/.NOAA/.NCEP/.EMC/.CMB/.GLOBAL/.Reyn_SmithOIv2/.monthly/).

### Calculation of body condition index

As differences in body mass may not only reflect energy reserves but also inter-individual differences in structural body size, we calculated a body condition index and determined between-year repeatabilities for both body mass and body condition. Separately for males and females, we tested for a correlation between either of the body size measurements that we had obtained (flipper length, bill depth and bill length) with body mass, and chose the measurement, for which the correlation coefficient was highest (bill depth in both sexes; Pearson R = 0.34, P < 0.001 and R = 0.31 and P < 0.001 for females and males, respectively). We therefore used bill depth and body mass to calculate the scaled mass index [63, 64] as a body condition index, separately for males and females.

### Statistics

We first tested for assortative mating according to body mass or body condition, running Pearson’s correlation tests between male and female body mass and body condition within pairs. We then calculated between-year repeatabilities for female and male body mass and conditions at egg laying for individual females and males that were included in the dataset at least in two years (N = 241 records of 76 females and 222 records of 78 males). As being paired with the same, or a different partner may affect the reproductive investment [32, 65], we calculated repeatabilities for A-egg mass and B-egg mass separately for individuals that remained with the same partner for at least two years (N = 185 records of 58 females and 64 males; numbers differed between sexes due to few cases of divorces) and those that paired with different partners for at least two years (N = 70 records for 31 females and 41 records for 20 males). Repeatabilities were calculated using restricted maximum likelihood (REML)-based linear mixed models as described in [66], using the rptR package [67] in R (version 3.10; [68]).

Subsequently, we tested the influence of environmental variables (explanatory variables) on body mass (dependent variables) by fitting linear mixed effects models to the complete dataset (N = 366 pairs). As several environmental variables were correlated with each other (e.g. SAM & SSTA: Pearson’s R = -0.11, P = 0.029) and to avoid collinearity, we decided against fitting several explanatory variables into a unique model but instead ran one model per explanatory variable, separately for female and male body mass. We included female or male identity, respectively, and year as random effects in models, and also ran a null model each. The best model (i.e. best explanatory environmental variable) was identified based on Akaike’s information criterion (AIC). In a final step, we tested the influence of female and male body mass (both as explanatory variables within the same model) on egg mass (A-egg mass and B-egg mass; as dependent variables in separate models) by fitting linear mixed effects models with female identity, male identity and year as independent random effects. When significant, we extracted the model estimates to present the effect sizes of female or male masses on A-egg mass or B-egg mass.

Models were fit with the lme4 package [69] in R, and all compared models were fit with restricted maximum likelihood (REML). We present t-values from model summaries. To obtain P-values, we compared the model with the variable in question and the null model, and only for this case based models on maximum likelihood (ML). We followed Nakagawa and Schielzeth [70] to calculate marginal R^2^ values (R^2^_m_, for the variance explained only by fixed effects) and conditional R^2^ values (R^2^_c_, based on the variance explained by both fixed and random effects).

## Results

### Repeatabilities of body mass, body condition and egg mass

Body mass and also body condition were significantly and highly repeatable in the same individuals across several years both in females and males (Table 1). Notably, there was no correlation between female and male body mass or body condition (Pearson’s R = -0.03, P = 0.547 for body mass, and R < 0.01 and P = 0.882 for body condition), indicating that birds did not pair assortatively according to body mass or condition.

**Table 1:** Repeatability estimates (± standard error) for body mass, body condition, A-egg and B-egg mass for individual females and males.

	Females	Males
**Body mass**		
all birds	R = 0.792 ± 0.035	R = 0.645 ± 0.058
**Body condition**		
all birds	R = 0.811 ± 0.031	R = 0.726 ± 0.045
**A-egg mass**		
same partner	R = 0.858 ± 0.029	R = 0.859 ± 0.034
different partner	R = 0.795 ± 0.070	R = 0.503 ± 0.175*
**B-egg mass**		
same partner	R = 0.803 ± 0.036	R = 0.810 ± 0.034
different partner	R = 0.703 ± 0.091	R = 0.519 ± 0.174

Repeatabilities for body mass and body condition were calculated for all birds that were recorded at least in two years (“all birds”: N = 241 records of 76 females and N = 222 records of 78 males). Repeatabilities for egg mass were calculated separately for birds that remained with the same partner for at least two years (“same partner”: N = 185 records of 58 females and 64 males) and for birds that paired with different partners for at least two years (“different partner”: N = 70 records for 31 females and N = 41 records for 20 males). P-values in all cases except for the one marked with an asterisk were highly significant (all P < 0.001)

*p = 0.002

Females were highly repeatable in their egg mass across years, independently of being paired with the same or with different partners (Table 1). Similarly, egg mass was highly repeatable for males that were paired with the same female partner for two or more years. When paired with different female partners, repeatability estimates for egg mass in males were markedly lower, albeit still highly significant (Table 1).

### Effect of environmental variables on body mass and subsequent effect of body mass on egg mass

**Fig 1:**
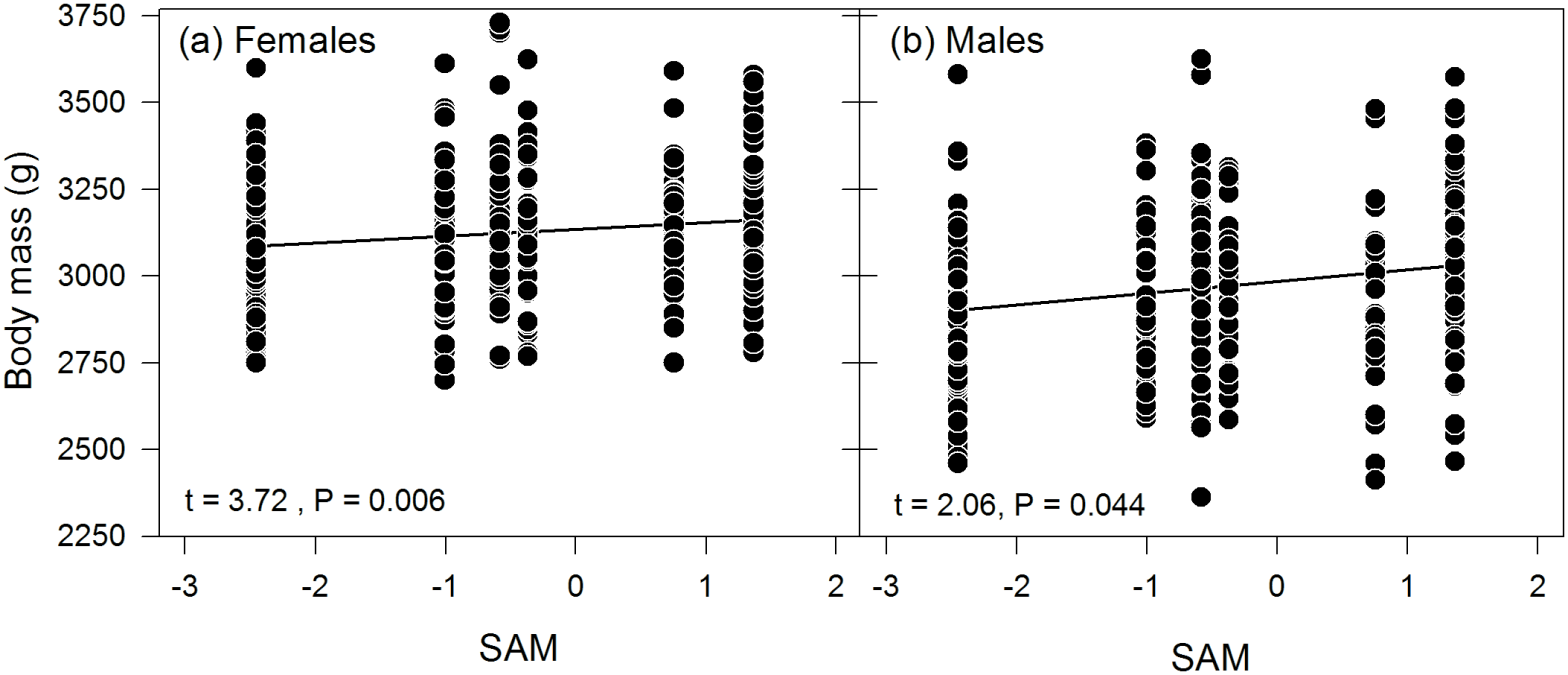
Adult body mass in response to Southern Annular Mode (SAM) for (a) female and (b) male southern rockhopper penguins. Regression lines show the direction of the relationship. t-values were obtained from the (REML-based) linear mixed effects models and P-values from likelihood ratio tests based on these models.

Models with different environmental variables to explain female and male body mass showed small to moderate differences in AIC (ΔAIC ≤ 3.7 within models for each sex; Table 2), and therefore small differences in overall explanatory power (c.f. R^2^_c_ values in Table 2). However, environmental variables clearly improved model fit compared to the null model (max. ΔAIC of 10.3 and 9.4 for females and males, respectively; Table 2). Amongst the environmental variables, SAM explained annual variation in both female and male body mass only slightly better than SSTA, while SOI in both sexes explained markedly less variation (Table 2). Body mass increased significantly under positive SAM and therefore colder conditions in both sexes (Fig 1; Models F1 and M1 in Table 2), while SSTA had no significant effect on either female or male body mass (both P ≥ 0.663; Models F2 and M2 in Table 2). While the environmental variables explained a relatively small proportion of variation in the models (at maximum 2.3% and 3.0% for females and males, respectively, as indicated by the R^2^_m_ values obtained from the model including SAM), the birds’ identity and year (both as random effects) contributed relatively more to the models’ fit as visible from the difference between R^2^_c_ and R^2^_m_ values (Table 2).

**Table 2:** Model outputs for the effect of candidate environmental variables and null models on female body mass (top) and male body mass (below) (= dependent variables).

Model	Dep. variable	Expl. Variable	Random effects	t	AIC	R^2^_m_	R^2^_c_
**F1**	**Female mass**	**SAM**	**F-ID + Year**	**3.72**	**4650.4**	**0.023**	**0.818**
F2	Female mass	SSTA	F-ID + Year	-0.38	4652.0	0.001	0.820
F3	Female mass	SOI	F-ID + Year	1.88	4654.1	0.015	0.819
Null	Female mass	-	F-ID + Year	-	4660.7	0.000	0.819
**M1**	**Male mass**	**SAM**	**M-ID + Year**	**2.06**	**4836.8**	**0.030**	**0.698**
M2	Male mass	SSTA	M-ID + Year	-0.05	4837.8	0.000	0.701
M3	Male mass	SOI	M-ID + Year	1.69	4839.2	0.024	0.702
Null	Male mass	-	M-ID + Year	-	4846.2	0.000	0.697

Year and either female identity (F-ID) or male identity (M-ID) were included as random effects. SAM = Southern Annular Mode, SSTA = local sea surface temperature anomaly, SOI = Southern Oscillation Index. R^2^_m_ values represent the variance explained only by fixed effects, and R^2^_c_ the variance explained by both fixed and random effects. N = 366 records for female and male body mass. All compared models were based on Restricted Maximum Likelihood (REML).

**Fig 2:**
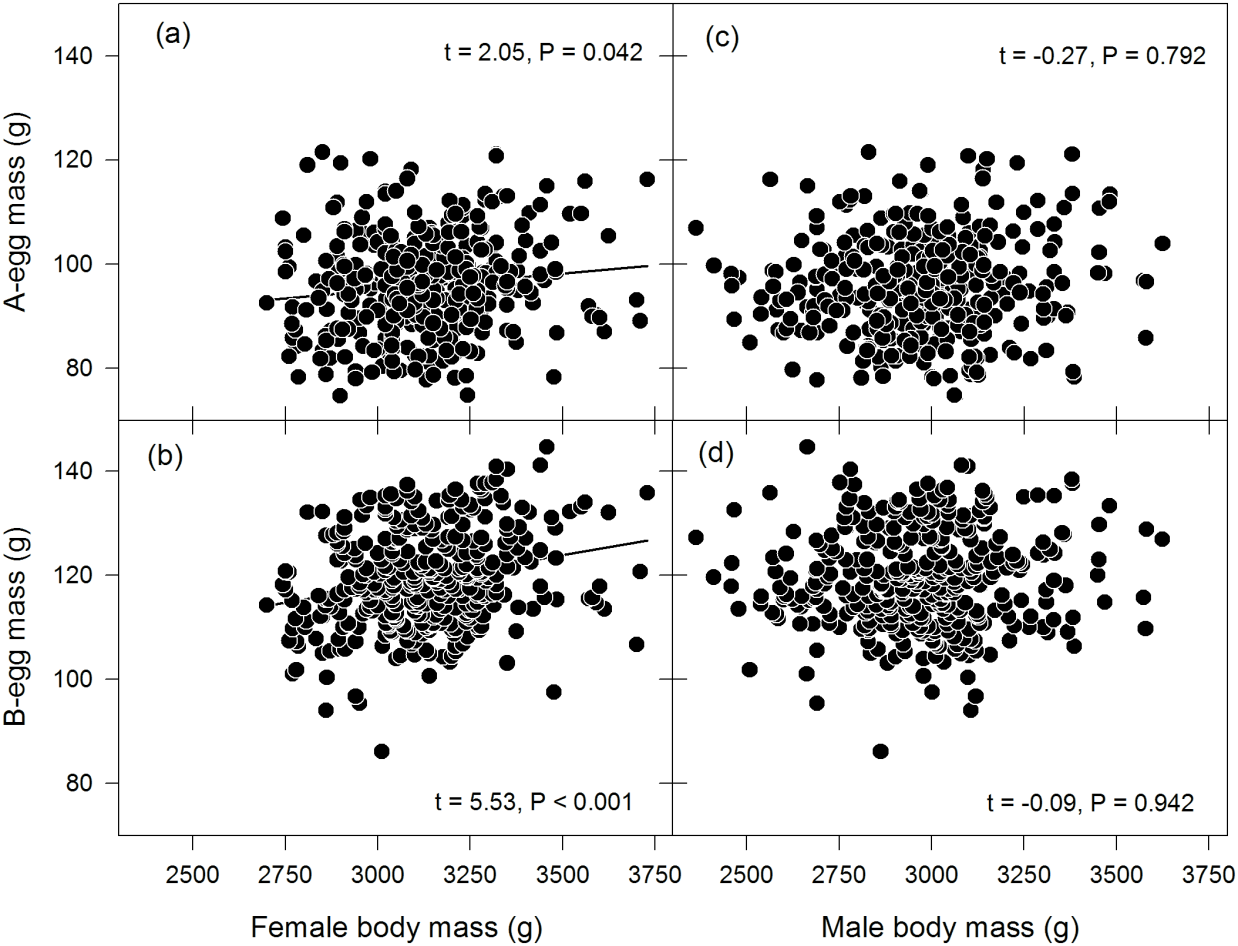
A- and B-egg mass in response to female (left) and male (right) body mass (both measured at or adjusted to clutch initiation date). Regression lines show the direction of the relationship (for significant effects only). t-values were obtained from the (REML-based) linear mixed effects models and P-values from likelihood ratio tests based on these models.

As expected, female body mass had a positive effect on both A- and B-egg mass (Fig 2), and therefore overall reproductive investment. The effect of female body mass on egg mass was stronger for B-eggs (t = 5.53, P < 0.001; estimated egg mass increase of 0.014 g ± 0.003 SE per g female body mass) than for A-eggs (t = 2.05, P = 0.042; estimated egg mass increase of 0.005 g ± 0.002 SE per g female body mass). Against our expectation, male body mass had no significant effect on either A-egg mass (t = -0.27, P = 0.792) or B-egg mass (t = -0.09, P = 0.942; Fig 2). Overall, female and male body mass together explained comparatively little variance in models as indicated by the small R^2^_m_ values (R^2^_m_ = 0.010 and 0.078 for A- and B-egg mass, respectively), whereas the birds’ identities and year (as random effects) explained relatively more (R^2^_c_ = 0.841 and 0.857 for A- and B-egg mass, respectively).

## Discussion

### Repeatability of body mass, body condition and egg mass

We found that across years both body mass and body condition were highly repeatable within the same females and males, indicating a high individual consistency of these traits in both sexes. We calculated body condition based on the scaled mass index, currently the best condition index based on mass-length data [64], and seen as a reliable indicator of body fat [64]. We therefore assumed that body condition of the birds reflected the energy reserves independently of the structural size of the bird. As repeatability estimates for body mass and body condition were similarly high, we infer that the repeatability in body mass was not primarily caused by consistent between-individual differences in structural size, but more likely caused by other effects. Possibly, the here observed consistent between-individual effects in body mass and reproductive investment could be linked to individually different foraging preferences. Rockhopper penguins are food generalists with a broad prey spectrum [34, 71]. In such generalist species, it is commonly observed that individuals specialize on specific prey or foraging areas [72, 73], which during winter [72, 74] could potentially explain the consistent between-individual effects in body mass and also reproductive investment.

Egg mass was also highly repeatable within females, and—to a lesser extent—within males, reflecting an individually consistent investment into egg mass especially in females. In view of the observed variation of body mass in response to Southern Annular Mode (SAM) across the years (see below), this result implies that adults showed a similar response to this environmental variation: Differences in body mass among individual birds were consistently maintained across years, while body mass of all individuals increased under positive SAM (reflecting colder conditions) and decreased under negative SAM (reflecting warmer conditions).

While structural body size and also body mass may be heritable, which automatically implies significant repeatability (e.g. [75, 76, 77]), estimates for actual repeatability of body mass within or across years have been rather rarely published. Our estimates for repeatability in body mass are in the same range as those for great tits (*Parus major*) measured in several winters [78], and weasels (*Mustela nivalis*) across a time span of up to 400 days [79], yet higher than in gray catbirds (*Dumetella carolinensis*) measured across several breeding seasons [80]. We obtained higher estimates for repeatability of body condition than were previously reported for breeding upland geese (*Chloephaga picta leucoptera*) [23], while this trait was not significantly repeatable across years in crimson finches (*Neochima phaeton*) [81]. To the best of our knowledge, this is the first study that presents repeatability estimates of body mass or body condition across one year or more in a seabird species.

For egg mass, repeatability estimates within females were in the same range as those from other studies (see review in [82]). As such, finding significant repeatability of egg mass in males that were paired with the same female over several years was expectable and has also been shown before (e.g. [65, 83]). Yet, considering that male body mass did not affect egg mass (i.e. females did not invest more into egg mass when paired with a heavy male; see discussion below), we were surprised to still find significant (albeit lower) repeatability estimates for egg mass in males when paired with different females. Indeed, our result contradicts a study by Phillips and Furness [65] that found clutch volume not to be repeatable for male Arctic skuas (*Stercorarius parasiticus*) paired with different females. On the other hand, Bańbura and Ziliński [84] found a similar result as ours, with average egg volume being repeatable for male barn swallows (*Hirundo rustica*) that paired with different females, and assumed assortative mating according to body condition as the underlying mechanism for this effect. In the framework of this study, we have shown that rockhopper penguins do not mate assortatively according to body mass or body condition (i.e. heavy males do not necessarily pair with heavy females). Nevertheless, other traits besides body mass or body condition may also play a role for reproductive investment, for example the timing of clutch initiation. In birds, early breeders usually lay heavier eggs than late breeders [23, 85]. As such, if rockhopper penguin males that occupy nest sites early mate consistently with early breeding females, and early breeding females lay heavier eggs than late breeding females, this could explain the significant repeatability for egg mass in males. We did not include clutch initiation date into analyses as the focus of this study was on body mass, and we recorded body mass on clutch initiation date. Therefore, investigating the relationships between body mass and clutch initiation date, or between clutch initiation date and egg mass would inevitably have raised problems with circularity.

### Phenotypic plasticity of body mass in response to environmental conditions

Out of the three temperature-related environmental variables that we included in our analysis to explain annual variation in adult female and male body mass, SAM showed a slightly better model fit than SSTA. SAM also showed a significant effect on body mass, while SSTA did not—although both variables were significantly negatively correlated. SAM is a broad-scale environmental variable that reflects climatic variability in the entire Southern Ocean [51]. The—relatively—better fit of this broad-scale environmental variable compared to the local one may be due to the fact that southern rockhopper penguins are migratory during winter (e.g. [86, 87]). They should therefore accumulate their energy reserves for reproduction at wintering grounds or during migration to breeding sites, and thus not in local waters around the Falkland Islands. This result is in line with earlier findings which show a relatively higher importance of broad-scale environmental variables on the breeding behaviour of migratory seabird species compared to resident species [59].

In agreement with our first prediction, body mass in both sexes increased under positive SAM values, which reflect lower sea surface temperatures and stronger westerly winds, and therefore overall colder conditions and higher primary productivity [88, 89]. SAM-associated climatic conditions (low SST and increased westerly winds) have already previously been associated with better foraging conditions and higher survival rates in the southern rockhopper penguin [48, 57, 90]. Furthermore, a recent study identified SAM also as the best explanatory environmental variable for clutch initiation date in rockhopper penguins, with birds breeding earlier under positive SAM and thus colder conditions [91]. The same study showed that total clutch mass in rockhopper penguins was best explained by local SST, and females laid heavier eggs under lower SST [91]. Within the marine food web, southern rockhopper penguins are located at a relatively low trophic level position compared to other seabird species [92], and are therefore likely to benefit rapidly from increased primary productivity. A positive SAM going along with low SST thus implies favourable foraging conditions for southern rockhopper penguins, and under these conditions, rockhopper penguins can reach higher body mass values—and lay heavier eggs. Notably, however, body mass differences among breeding seasons were not very distinct (Fig 1), and SAM explained at maximum 3% of the variation of body mass in models, thus much less than individual effects. The overall phenotypic response to SAM in the here analysed time period therefore appeared rather limited, and inter-annual variation in body mass was lower compared to data presented by Crawford et al. [29, 93]. These differences between our study and those by Crawford et al. [29, 93] could, however, be caused by the longer time series and—inevitably—larger environmental variability during their study period. Alternatively, regional differences in the environmental conditions and/or food availability in wintering grounds of rockhopper penguins from Marion Island could be less predictable than those of rockhopper penguins from New Island leading to more variation in body mass among years.

### Effect of body mass on egg mass

In agreement with the second prediction, A-egg mass and B-egg mass, and consequently overall reproductive investment increased with female body mass. Notably, this effect was stronger for B-eggs than A-eggs. As chicks from A-eggs rarely fledge, they may rather form an “insurance” for the case that the B-egg or chick is lost [37, 94]. Thus, by investing relatively more into B-eggs when being heavy, females selectively increased their investment into those eggs that would most likely produce fledglings while keeping “insurance costs” low. This also agrees with previous findings from the same population of rockhopper penguins which showed that females decrease their relative investment in A-egg mass compared to B-egg mass with increasing (later) laying dates [91].

In contrast to the effect of female body mass, we could not confirm an effect of male body mass on egg mass—despite the significant repeatability estimates of egg mass for males. These findings are concordant with the literature, in which positive effects of female body mass, body size or a body condition index on egg mass or egg size are well described (e.g. [17, 95, 96]). A significant relationship between male mass and egg mass has been shown less frequently (but see [17]), even when other related parameters like testis size were positively correlated with egg mass [19]. In line with this and our current results, Poisbleau et al. [32] did not find an effect of male body mass on total clutch mass (i.e. A-egg mass + B-egg mass) either. Thus, this longer-term study confirmed that female rockhopper penguins do not adapt their overall reproductive investment when paired with a heavy male partner.

## Conclusions

We here showed that—despite substantial intra-annual body mass variation—adult female and male body mass at breeding is highly repeatable across several years in rockhopper penguins. Furthermore, we showed that SAM affected adult body mass, with penguins being heavier under colder conditions, and heavier females producing heavier clutches. Relating our current knowledge to the warming trend of atmosphere and ocean, we can expect that cold environmental conditions that favour high food availability and consequently high body mass and high egg mass in rockhopper penguins (positive SAM) will occur less frequently in the future [2]. Thus, we might observe a long-term phenotypic response to lower body mass and lower egg masses in rockhopper penguins (also see [76]). We might furthermore expect that other long-lived species that also rely on the marine food web, especially within the group of seabirds, will in the long-term show similar phenotypic responses.
